# Tuning Enzymatically Crosslinked Silk Fibroin Hydrogel Properties for the Development of a Colorectal Cancer Extravasation 3D Model on a Chip

**DOI:** 10.1002/gch2.201700100

**Published:** 2018-05-24

**Authors:** Mariana R. Carvalho, Fátima Raquel Maia, Sílvia Vieira, Rui L. Reis, Joaquim M. Oliveira

**Affiliations:** ^1^ 3B's Research Group – Biomaterials Biodegradables and Biomimetics University of Minho Headquarters of the European Institute of Excellence on Tissue Engineering and Regenerative Medicine Avepark 4805‐017 Barco Guimarães Portugal; ^2^ ICVS/3B's – PT Government Associate Laboratory Braga/Guimarães Portugal; ^3^ The Discoveries Centre for Regenerative and Precision Medicine Headquarters at University of Minho Avepark 4805‐017 Barco Guimarães Portugal

**Keywords:** 3D model, colorectal cancer, extravasion, microfluidics, silk

## Abstract

Microfluidic devices are now the most promising tool to mimic in vivo like scenarios such as tumorigenesis and metastasis due to its ability to more closely mimic cell's natural microenvironment (such as 3D environment and continuous perfusion of nutrients). In this study, the ability of 2% and 3% enzymatically crosslinked silk fibroin hydrogels with different mechanical properties are tested in terms of colorectal cancer cell migration, under different microenvironments in a 3D dynamic model. Matrigel is used as control. Moreover, a comprehensive comparison between the traditional Boyden chamber assay and the 3D dynamic microfluidic model in terms of colorectal cancer cell migration is presented. The results show profound differences between the two used biomaterials and the two migration models, which are explored in terms of mechanical properties of the hydrogels as well as the intrinsic characteristics of the models. Moreover, the developed 3D dynamic model is validated by demonstrating that hVCAM‐1 plays a major role in the extravasation process, influencing extravasation rate and traveled distance. Furthermore, the developed model enables precise visualization of cancer cell migration within a 3D matrix in response to microenvironmental cues, shedding light on the importance of biophysical properties in cell behavior.

## 1 Introduction

Colorectal cancer (CRC) is a major cause of morbidity and mortality worldwide, and accounts for over 9% of all cancer incidence. It is the third most common cancer worldwide and affects men and women equally.^1^

In order to win the battle against cancer, further advances are in great need to unveil identification of cancer‐causing agents in in vitro and in vivo animal models, as well as for the development of personalized therapies, drug screening, and to provide insightful knowledge on the mechanisms of tumor growth and metastasis.^2^ Although in vivo animal models comprise the complexity of the metastatic cascade in a living system, visualization of the distinct events is nearly impossible. In fact, recent studies suggest that the correspondence between animal models and successful clinical trials does not reach 10%.^3^ Furthermore, animal models do not allow control of cell–cell and cell‐extracellular matrix (ECM) interactions, making it difficult to really understand the role of each stromal component in the tumorigenesis process. On the other hand, 2D in vitro models have reduced physiological relevance, capturing only limited aspects of the tumor microenvironment.^4^ Therefore, 3D models, comprising the integration of tissue engineering (TE) strategies with microfluidic technologies have sparked a breakthrough into the design of in vitro microfluidic culture models. These better adapt to morphological changes in tissue structure and function over time, providing a level of precision control that could not be achieved before.^5^ Microfluidics can provide useful model systems to investigate complex phenomena under combination of multiple controllable biochemical and biophysical microenvironments, coupled with high resolution real time imaging.^6^, ^7^

This type of strategy does provide a powerful tool to develop more in vivo—like settings that can be applied to study cancer. Special focus on model dimensionality and microenvironment complexity is given, as a significant amount of experimental evidence has shown that both mechanical and chemical stimuli from the cell microenvironment play a key role in several types of cell behavior, namely in terms of migration.^8^ Migration is frequently used as broad term in biology and applies to any directed cell movement, even in a 2D setting.^9^ The ability to migrate allows cells to change their position within tissues or between different organs. However, in pathology, invasion is defined as the crossing of (tissue) 3D barriers, into the underlying interstitial tissues by malignant tumor cells.^9^

So far, several synthetic and natural hydrogels have been used as ECM—like materials in the in vitro cell culture studies, offering characteristics such as biocompatibility and bioactivity, and have undoubtedly been proven to influence cells' behavior and fate.^10^, ^11^, ^12^ However, the full potential of these recent in vitro models in research and therapy has remained unrealized, owing to the poorly defined animal‐derived matrices in which cells are grown and in particular, mostly limited to use of Matrigel.^13^ Trying to address this issue, Gjorevski et al. used polyethylene glycol (PEG) hydrogels to define the microenvironmental parameters that govern organoid formation.^12^ That study enhanced our understanding of the mechanical regulation of intestinal stem cells by considering the effects of matrix stiffness, which had not been examined before, as performing controlled mechanical modulations in vivo and in Matrigel is challenging. The authors observed that intestinal stem cell expansion was optimal within matrices of intermediate stiffness (1.3 kPa), but that organoid formation in low stiffness matrices was optimal (150 Pa).^12^

In our study, applying these concepts, we propose the use of a 3D dynamic model using a new biomaterial developed by our group: Horseradish peroxidase crosslinked silk fibroin (eSF) hydrogels.^14^ eSF hydrogels easily allow the tuning of mechanical properties and identify the most suitable stiffness to study cancer cell migration. Silk is a famous natural fiber produced by the silkworm (*Bombyx mori*) cocoons and is composed of two types of protein (fibroin and sericin), lacking the common motifs such as laminin and collagen. For this reason, silk allows the substrates' mechanical contribution to cell fate to be isolated better than chemically bioactive materials, such as Matrigel. Therefore, we are able to better define and modulate the key ECM parameters that govern colorectal cancer cell migration.

By its turn, Matrigel is used as a control, since it has already been established as 3D substrate for modeling cancer microenvironments.^15^, ^16^ Moreover, cell migration in this new platform is compared to traditional 3D Boyden chamber invasion assay. Regardless of their vast value, they do not provide tight control over the local environment, lack the ability to precisely control the spatial organization of cells in 3D matrices, cell–cell and cell–ECM interactions. Imaging is rather limited, disclosing the 3D microfluidic in vitro models' physiological relevance. The evaluation of the effect of hVCAM‐1 was also assessed in both invasion models. It is now known that VCAM‐1 is involved in mediating tumor cell adhesion to vascular endothelial cells and promoting the metastatic process.^17^ Also, serum concentrations of ICAM‐1 and VCAM‐1 are significantly elevated in the patients with colorectal cancer in comparison with a group of healthy subjects.^18^ One of the possible mechanisms is that the rolling cancer cells become activated by locally released chemokines present at the surface of endothelial cells. This triggers the activation of integrins from the cancer cells allowing their firmer adhesion to members of the Ig‐CAM family such as ICAM and VCAM‐1, initiating the transendothelial migration and therefore the extravasation process.^17^ VCAM‐1 is therefore thought to play a key role in the process of malignant progression and its presence studied in terms of impact on cell migration.^19^

In this work, different concentrations of eSF hydrogels were produced as 3D matrices and fine‐tuned in terms of mechanical properties by means of varying the concentration of silk solution between 1% and 3%. The realization of a 3D CRC model was achieved by using a commercially available microfluidic chip (Vena4, Cellix) suitable for intravasation and extravasation assays comprising a 3D microwell for the testing of ECM like materials, together with HCT‐116 cancer cells. Cell migration studies using this 3D dynamic microfluidic platform in different conditions, as well as the traditional modified Boyden chamber were carried out up to 48 h days of culturing. Matrigel was used as control. Due to its importance during the metastization of colorectal cancer cells, hydrogels were supplemented with the vascular adhesion molecule hVCAM‐1 to validate our model.

## 2 Results

### 2.1 Characterization of Hydrogels

#### 2.1.1 Mechanical Properties Determination: Rheology

The influence of polymer type and concentration of the used hydrogels was studied in terms of their rheological behavior in order to analyze the mechanical properties of the hydrogels. Stress sweeps (0.1 Hz) were first performed to determine the linear viscoelastic region (LVR) for all the tested conditions. Frequency sweeps (0.01–10 Hz) were then performed within the LVR. The values of the shear storage modulus (*G*′) and loss modulus (*G*″) are presented in **Table** **1** and were obtained at a frequency of 0.1 Hz.

**Table 1:** Composition, rheological properties, and original mesh size of hydrogels at a frequency of 0.1 Hz.

Name	Cells	Incubation time	Storage modulus[*G*′, Pa]	Loss modulus [*G*″, Pa]
1% eSF	No	30 min	65 ± 9	8 ± 5
2% eSF	No	30 min	488 ± 72	16 ± 4
3% eSF	No	30 min	1136 ± 94	20 ± 3
Matrigel	No	1 h	45 ± 15	4 ± 0.5

Storage modulus *G*′ is known as the “solid‐like” or elastic component of the gel, and *G*″ (Loss modulus) as the “liquid‐like” or viscous component. The system is considered a gel if the value of *G*′ is greater than *G*″, which was confirmed by the results in Table 1, where all the values for the loss modulus are many folds smaller than the storage modulus, which was expected for a hydrogel system that is stable during time. The higher the *G*′, the less it deforms under compression and the more energy it can retain and store.

Indeed, when comparing the *G*′ value between eSF hydrogels and Matrigel, the latter is much lower, corroborating the fact that even after crosslinking, Matrigel is difficult to handle and is not a consistent material, which is an important feature when considering performing further studies on the hydrogels. When comparing eSF hydrogels, the *G*′ of 3% eSF hydrogel is much higher than the 2%, which, in turn is much higher than the 1% formulation. Consequently, rheology measurements confirm that the stiffness and mechanical properties of the hydrogels increase with increasing polymer concentrations in the case of silk fibroin, and this is due to the increase in the number of polymer chains and crosslinking points within the system, allowing the tuning of mechanical properties and further study of possible applications in cell migration and tumor/spheroid formation.

### 2.2 Outward Cell Migration (Modified Boyden Chamber Assay)

**Figure 1:**
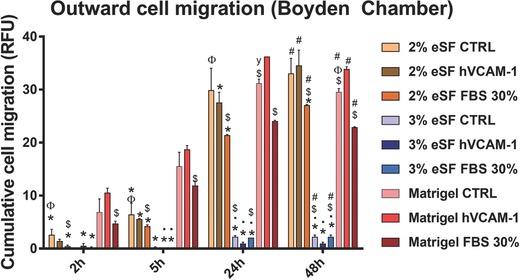
Quantification of outward migration of HCT‐116 (labeled with Red Cell Tracker in modified Boyden chamber from 2% and 3% eSF hydrogels and Matrigel coating, in response to the presence of hVCAM‐1 and 30% of Fetal Bovine Serum (FBS). (* indicates significant differences when comparing to Matrigel coating at each time point; • indicates significant differences when comparing to 2% eSF at each time point; $ indicates significant differences when comparing to VCAM at each time point; (Φ) indicates significant differences when comparing to FBS at each time point; # indicates significant differences when comparing to time point 2 h.

This model was chosen to do a comparative study of colorectal cancer cells' behavior regarding cell migration. As performed in our 3D dynamic model, 2% and 3% eSF and Matrigel were used and the presence/absence of hVCAM‐1 in order to have comparable data. Besides, another condition was added in this experiment: a gradient of serum (from 0% to 30% FBS), generally used as chemoattractant in the modified Boyden chamber assay, to be compared with migration observed with the gradient of hVCAM‐1. As this molecule is thought to be involved in colorectal cancer migration and metastasis, it is interesting to compare it with FBS. In the condition described as the control, the upper and lower chambers are filled with normal Dulbecco's modified Eagle medium (DMEM) medium with no molecules' gradient. Regarding the hydrogels, 10 µL of 2% and 3% eSF hydrogels were crosslinked at 37 °C on top of the membrane. This is the minimum quantity to cover the surface of the membrane. For Matrigel, manufactures' protocol was followed and a dilution of Matrigel was used to produce a thin coating. Different time points were analyzed to see the progression of migration and corroborate the fact that this kind of phenomena occurs mainly within the first 24 h, as can be seen in **Figure** **1**.^20^, ^21^

Also, to prevent the quantification of cell proliferation instead of cell migration, the experience was terminated at 48 h.

Figure 1 shows the final results of cumulative cell migration (in terms of fluorescence quantification) from 2 up to 48 h. Fluorescence at 0 h was considered our blank. The first noticeable result is that migration phenomena starts almost immediately after the seeding on top of the hydrogels and that the number of cells crossing through the hydrogel and the membrane toward the bottom chamber grows until 48 h of culturing. At 48 h, by the end of the experience (the same time point that is going to be analyzed in 3D model Vena4), and where cell migration is more evident, interesting results can be withdrawn. Both in the case of 2% eSF and Matrigel, there is an intense cell migration in the presence of hVCAM‐1, significantly higher than in the presence of the traditional chemoattractant FBS and the control (absence of any chemoattractant). Interestingly, cell migration to the bottom side of the membrane increases continuously until 48 h, as all the conditions are significantly different when comparing to time point 2 h. Maybe one of the most relevant observations in this experiment is that from the beginning, especially at first time points, Matrigel allows more cell migration than 2% and 3% eSF. Alternatively, by the last time‐points, there is no substantial difference in the fluorescence intensity between 2% eSF and Matrigel. At last, it becomes clear that 3% eSF allows very little cell migration when comparing to 2% eSF and Matrigel.

**Figure 2:**
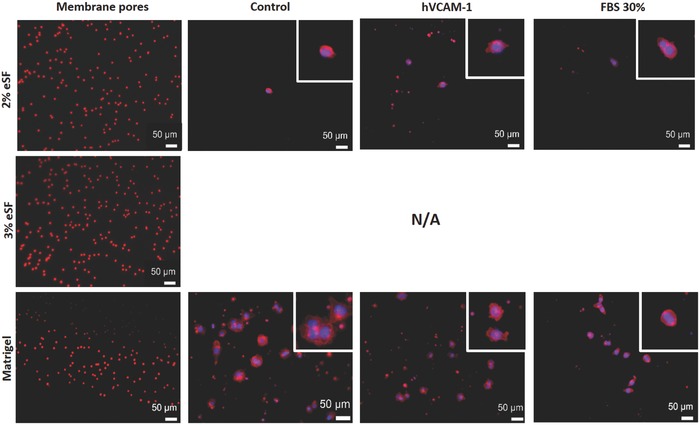
Representative panel of outward migration of HCT‐116 cells from 2% and 3% eSF and Matrigel coating in response to gradients of hVCAM‐1 and FBS. Migrating cells attached to the lower side of the insert membrane at 48 h stained with DAPI (blue) and phalloidin (Red). The first column represents the pores of the membrane without cells. N/A denotes no cell migration.

The panel represented in **Figure** **2** shows the cells that have migrated thought the hydrogels and across the black membrane of the modified Boyden chamber. Initially, cells were prelabeled with red cell tracker for the quantification analysis, but for better visualization, they were later stained with 4,6‐diamidino‐2‐phenylindole (DAPI) (blue‐nucleus) and phalloidin (red—f‐actin filaments) and observed under inverted fluorescence microscopy. The first column of the image shows every condition, but without cells. Therefore, the pores of the membrane are clearly visible under red fluorescence and no cells are observable. The second column, described as control, corresponds to the condition in which the upper and lower chambers are filled with normal DMEM medium with no molecules' gradient. Corroborating the results shown in the previous graphic, no cells were observed in the bottom of membranes with 3% eSF hydrogels. In the case of 2% eSF hydrogel and Matrigel coating, it is possible to observe that the cells have migrated and present their normal round shape morphology.

### 2.3 In Vitro Studies under a 3D Microfluidic Platform

#### 2.3.1 Cell Migration Assay in 3D VenaT4 Biochips

**Figure 3:**
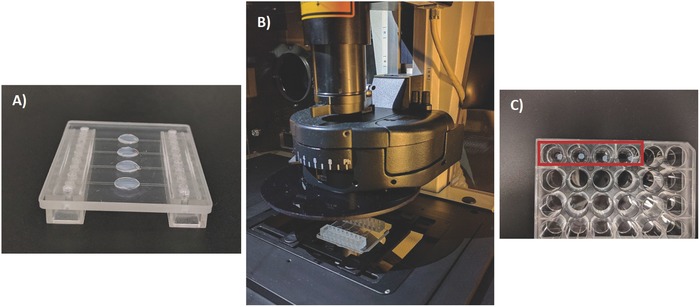
A) Example of Vena4 chip with eSF hydrogels placed on the microwells. B) Vena4 placed under the confocal microscope for migration studies. C) Retrieval of eSF hydrogels out of the microfluidic chip for further analysis.

Although there is still a lot of uncertainty regarding the critical step in the formation of metastatic tumors, the ability of circulating tumor cells to adhere to and transmigrate across the endothelium at a remote site is certainly essential. In the case of this experience, the extravasation is mimicked when adhered red labeled HCT‐116 cells seeded on the microchannel and subjected to flow perfusing media, cross the 8 µm pore membrane and migrate toward the underlying hydrogel (from top down) and observed on the confocal microscope, as can be seen in **Figure** **3**.

**Figure 4:**
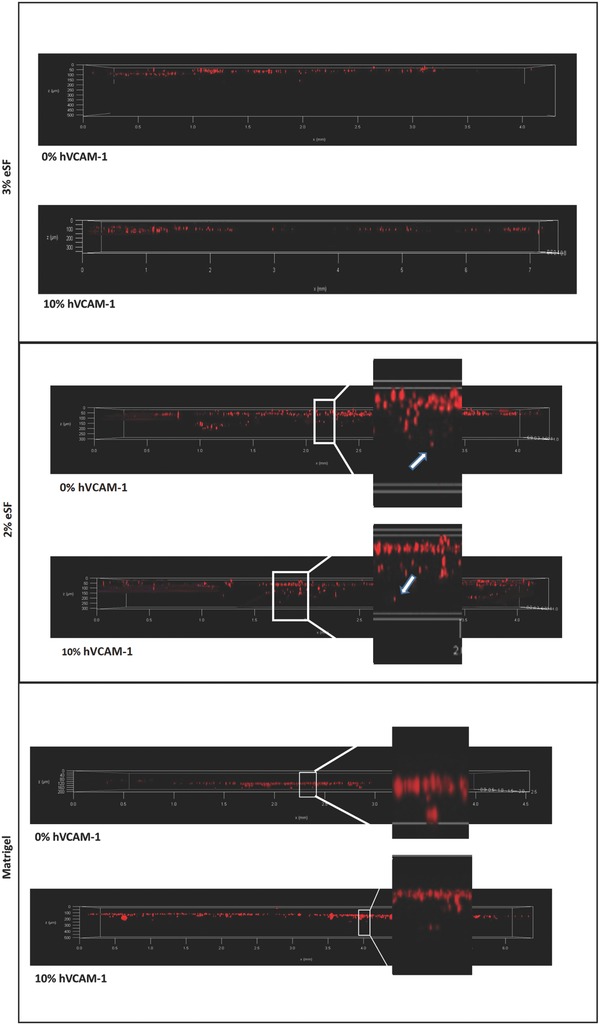
Representative confocal microscopy images of red‐labeled HCT‐116 cells' migration toward 3% and 2% eSF hydrogels and Matrigel, (with and without hVCAM‐1) in microfluidic chip Vena4 at 48 h.

In this case, pre‐crosslinked 2% and 3% eSF and Matrigel hydrogels are placed in the microwell under the microchannels. Since literature suggests this type of migration occurs within the first 24 h of experience, all of our experiments lasted up to 48 h.^21^, ^22^ Examples of renderings of 3D confocal stacks of all hydrogels are shown in **Figure** **4**. Confocal microscopy images were taken 3 h after cell seeding, in order to let them adhere to the microchannel in the 3D model (data not shown), and no migration was observed in any of the tested formulations. This means that all of the labeled cells were within the microchannel. After 48 h of experience, we placed the microchip in the confocal microscope and assessed cell migration. When testing eSF hydrogels as ECM‐mimicking alternatives, in the 3% formulation no cell migration appeared to happen, even in the presence of hVCAM‐1. However, when observing migration experiments with 2% formulation, results show a surprising difference and a large number of cells had crossed the membrane and migrated toward the center of the 2% eSF hydrogel. Moreover, in the case of 2% eSF, in the absence of hVCAM‐1, we observed cells migrating about 100–150 µm in depth. On the other hand, in the presence of the hVCAM‐1, cells could migrate a larger distance, up to 200 µm (see white arrow in Figure 4).

Regarding our control material, Matrigel, as depicted by the representative Figure 4, a few cells crossed the membrane and migrated through the microporous membrane toward the Matrigel. No apparent exacerbation in terms of cell migration was observed in the presence of hVCAM‐1 molecule.

Summarizing the results of cell migration in the 3D microfluidic chip, we observed that when testing eSF, the softer hydrogel (2%) allowed increasing levels of cell migration, and within this condition, the presence of hVCAM‐1 was translated in a larger distance traveled by the cells, inside the hydrogel. However, concentrated Matrigel hydrogel, generally used in these types of experiments, do not favor cell migration and no effect of hVCAM‐1 is visible.

#### 2.3.2 Live/Dead Assay

**Figure 5:**
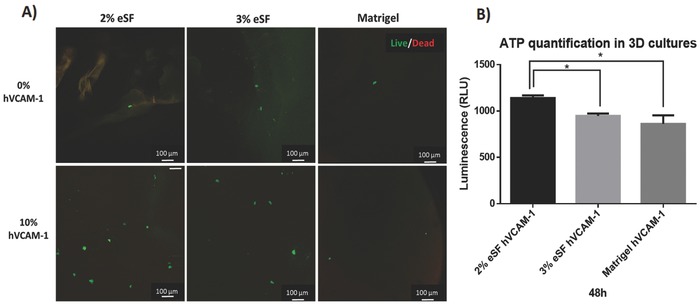
A) Cell viability (live/dead assay) of HCT‐116 cells that migrated in the hydrogels after 48 h of culture in Vena4 microfluidic chip. B) Cell viability of 3D cultures after 48 h of culture (2% and 3% eSF and Matrigel hydrogels with hVCAM‐1). Data are presented as mean ± stdev (*n* = 3), (*) denotes statistical differences (*p* < 0.05).

After assessing HCT‐116 colorectal cancer cellś migration from the microchannels toward the hydrogels in the Vena4 chip after 48 h in culture, we wanted to qualitatively determine cell viability. For that, the same experimental set up was used, but without prelabeling the cells. After 48 h, the hydrogels were carefully removed from the Vena4 microfluidic chip (Figure 3C) and live (green) and dead (red) cells were stained by adding Calcein‐AM/Propidium Iodide (PI) and hydrogels observed under confocal microscopy. Calcein‐AM fluoresces in green in case of living cells, as it is cleaved by esterases and produces green fluorescence, which is confined in the living cells by an intact cytoplasmatic membrane. PI is red as it is able to enter dead cells and nuclei through the damaged membrane and binds to fragmented DNA, emitting a red fluorescence. As observed in the representative panel in **Figure** **5**A, all the cells that have migrated toward the hydrogels in all the conditions were still viable. Although this is not a quantitative assay, it is worth noticing a higher number of (viable) colorectal cancer cells in hydrogels supplemented with hVCAM‐1, and even more pronounced in 2% eSF, corroborating the confocal images results seen before in Figure 4. This means that this 3D dynamic model allows for cell viability. The hydrogels are kept hydrated, although they are not submerged in medium, just subjected to media perfusion on top of the microchannels.

#### 2.3.3 Adenosine 5'‐triphosphate (ATP) Quantification

After the extravasation experiments with Vena4, where the goal was to assess the best formulation to observe cell migration from the microchannel to the underlying hydrogel (confirmed through confocal microscopy), we wanted to quantitatively determine cells viability in the hydrogels. Cells' viability inside the hydrogels is an important parameter to determine their suitability as ECM‐like matrices. For that, the extravasation experiment was carried out during 48 h, after which the hydrogels were retrieved from the microfluidic chip and tested for bioluminescent‐based ATP quantification. The reaction was catalyzed by the enzyme luciferase obtained from the firefly (*Photinus pyralis*). The MgATP2‐ converts the luciferin into a form which is capable of being catalytically oxidized by the luciferase in a high quantum yield chemiluminescent reaction. Moreover, a calibration curve using as low as five cells was used to determine its correlation with bioluminescent signal in 2D (Supporting Information). Although we cannot directly correlate the number of cells obtained in 2D and 3D, the signal obtained when testing the hydrogels show that the cells remained viable, corroborating the previously showed live/dead assay results, as can be seen in Figure 5B. Also, it was possible to see that, in agreement to what was observed by confocal microscopy, the extravasation experiment performed with 2% eSF shows the strongest signal, being significantly different from the 3% formulation and Matrigel.

## 3 Discussion

It is now more recognized than ever that the integration of microfluidic techniques together with advanced biomaterials such as ECM mimicking tunable hydrogels can supply a unique platform to develop 3D organized constructs mimicking the in vivo tumor microenvironment.^23^, ^24^ Concerning this, it was our goal to take advantage of our group's great expertise in biomaterials and test eSF hydrogels in the context of colorectal cancer metastasis. Silk is an excellent candidate for cancer related studies due to its biocompatibility and highly tunable properties.^14^ To the best of our knowledge, there has been lacking specific attempts to use tunable silk hydrogels as 3D ECM like materials to push forward the field of colorectal cancer metastasis research, combined with a confocal compatible microfluidic platform.

This innovative work aims at using a dynamic 3D microfluidic platform to test several silk hydrogel formulations for HCT‐116 cell migration (extravasation). Also, an extensive comparative study between the traditional modified Boyden chamber assay and our 3D microfluidic chip to assess colorectal cancer cell invasion, using 2% and 3% eSF hydrogels and Matrigel as a control group was performed. Although 1% eSF hydrogels were produced and characterized in terms of rheology, they were not tested for cell migration, as the 2% silk formulation was the highest formulation to allow HCT‐116 cancer cell migration in a dynamic environment, and robust enough to retrieve for further analysis such as ATP quantification and live/dead (Figure 3). Moreover, the influence of hVCAM‐1, a molecule widely present in colorectal cancer metastasis processes is also assessed in order to validate our 3D dynamic model.^17^

An innovative aspect of our study is that in our proposed 3D model, unlike most models, which focus on seeding cells on hydrogel sheets as well as encapsulating them within the 3D matrices, we seed cells on a microchannel, allow them to adhere, and observe their migration through a microporous 8 µm membrane and toward the hydrogel.^25^ Therefore, this extravasation model represents a more realistic scenario of the extravasation process.^26^ Literature suggests metastasis assays, being intravasation or extravasation, generally occurs within the first 24 h of tumor cell contact with the endothelium, therefore we decided to study this phenomena within the first 48 h.^27^

Based on the widely used modified Boyden chamber, we determined cell invasion by quantifying the outward cell migration of fluorescent labeled cells. In this assay, a layer of ECM like matrix is deposited on the porous membrane. Although collagen and laminin are also used, this layer is typically a Matrigel coating.^28^ Results of migration show that migration rates are similar between 2% eSF and Matrigel at 48 h, with a significant increase when in the presence of hVCAM‐1 in both cases. This result is relevant because it shows differences between hVCAM‐1 and FBS gradients, being the latest, almost always used as chemoattractant.^9^ It exposes the real influence of mimicking the in vivo microenvironment cues when studying cancer cell migration. Regarding the 3% eSF conditions, it becomes clear that the matrix does not favor cell migration in the Boyden chamber assay. In addition, in the first hours of the assay, results were striking in terms of the difference between cancer cell migration in Matrigel and 2% eSF, showing that Matrigel coating allows significantly more migration than 2% eSF.

When moving to our newly developed 3D microfluidic model, results are outstandingly different. As can be seen in Figure 4, after 48 h of dynamic culture, when analyzing the tissue engineered 3D model in the confocal microscope, no noticeable cell migration was perceived, and no observable effect was attributed to the presence of hVCAM‐1 in the 3% eSF hydrogel. When moving to the 2% formulation, we observed it allowed for a large number of cells to move from the microfluidic channel, through the microporous membrane and into the hydrogel. In the case of 2% eSF hydrogel crosslinked in the presence of 10% hVCAM‐1, it was translated in a greater distance traveled by the cells. More quantitative assays are needed to corroborate this information, but the representative confocal images leave no doubt about this change of behavior and add evidence on the association of hVCAM‐1 with malignant potential in colorectal cancer. On the other hand, when comparing the observations in the two tested scenarios (3D static (modified Boyden chamber assay) and 3D dynamic (our proposed microfluidic model)), a major difference is detected in cell behavior in the case of our control material: Matrigel. Whereas in the Boyden chamber cells migrate in large numbers from the first hours in the Matrigel coating (Figure 2), in the dynamic 3D model, this does not happen (Figure 4). These findings came as a surprise when analyzing the rheology results of the hydrogels. In fact, considering the data from rheology analysis it was expected that 2% eSF, (*G*′ value of 488 ± 72 Pa) allowed less cell migration than Matrigel (*G*′ value of 45 ± 15 Pa), since it is stiffer. Nevertheless, the opposite trend was observed, with more cell migration in the stiffer 2% silk hydrogel than the softer Matrigel. These phenomena could be explained by the different composition of the hydrogels. In this sense, silk hydrogels has no binding motifs (e.g., Arginylglycylaspartic acid (RGD) peptide), but Matrigel has natural mammalian binding motifs. It has already been discussed and reported in the literature a possible biphasic role of Matrigel that can partially explain these phenomena: at low concentration, such as coating, Matrigel facilitates migration, most probably by providing a supportive and growth factor retaining environment. At high concentration, Matrigel slows down migration, possibly due excessive attachment.^22^ In the Boyden chamber assay, following suppliers instructions, we used a Matrigel coating by diluting Matrigel to 250 µg mL^−1^, nearly 20‐fold less concentrated then the hydrogels used in our 3D dynamic model.

On the other hand, silk hydrogels obtained from *Bombyx mori* are not completely deprived of mammalian binding motifs. Silk proteins are comprised of a main heavy chain, which can be considered a hydrophobic protein with coblock design.^29^ Hydrophilic segments are involved in the self‐assembling process, resulting in changes in water content and regulation of the mechanical properties of the final material, which can explain cell migration in these matrices.^29^ In addition, two short amino acid sequences that are RGD‐like are present in the N‐terminal segment of heavy‐chain, VTTDSDGNE and NINDFDED, as well as the recognized fibroblast integrin.^29^

In fact, Friedl et al. described two different migration movements, mesenchymal and amoeboid migration.^30^ In the first, mesenchymal migration, cells exhibit high attachment and cytoskeletal contractility, which allow cells to migrate. But in the second, amoeboid migration, cells present poor attachment and a lack of stress fibers, which results in a round morphology. For so, they develop a strategy that consists in the formation of blebs and the use of propulsive forces to migrate. In this sense, the 2% eSF matrices possess mechanical properties that can allow amoeboid movements.^30^ In the case of 3% matrices, similar results were observed when comparing the Boyden chamber assay to the proposed 3D dynamic model. The resistance to cell migration in the 3% eSF formulation, even in the presence of chemoattractant, can be explained by matrix stiffness. By its turn, a *G*′ value of 1136 ± 94 Pa of the 3% silk formulation is simply too stiff to allow cell migration through the polymer's fibers, as confirmed by Bott et al., who revealed that in spite of matrix sensitivity to proteases (e.g., MMP) and the presence of cell‐integrin binding sites, at high stiffness (*G*′ > 1200 Pa) the matrix acts as a barrier for cells cultured in 3D to migrate.^31^ As a matter of fact, these results are in alignment with those observed by Gjorevski et al., showing that in the case of PEG hydrogels, a higher matrix stiffness of 1300 Pa is suitable for cell expansion, but organoid development is optimal in a later stage, in gels with a lower stiffness of around 100 Pa, which can be achieved by the presence of metalloproteinases.^12^ In our case, we are interested in studying cell migration, a different phenomenon than cell expansion and organoid formation, where the intermediate stiffness seems to work best. In a further stage, by using longer periods of culture in dynamic conditions, we aim at creating a tumoroid by modulating silk fibroin hydrogels mechanical properties to achieve lower stiffness, such as the 1% eSF formulation (64 ± 9 Pa, Table 1).

It is important to bear in mind that the effects of ECM properties on the normal epithelial morphogenetic program are fundamentally different from those reported for cancer cells in synthetic gels, most likely reflecting central differences between developmental morphogenesis and tumorigenesis.^32^ Matrix stiffness is therefore a crucial biophysical aspect of the tumor microenvironment and, consequently, has been profoundly studied in collagen, polyacrylamide, and Matrigel hydrogels, but not in silk.^33^ Therefore, the proposed 3D dynamic model offers an opportunity to better understand the role of mechanical properties of the matrix environment, assessing specific effects of matrix stiffness on colorectal cancer progression.^33^, ^34^

Taken together, these facts can explain the differences we found between the traditional static and dynamic models and disclose the importance of using relevant in vitro models.

After our study, we believe we are facing a question of developing a microenvironment mimic material, ultimately choosing between bio‐based versus polymer‐based ECM like materials. Although there is undoubtedly a growing number of studies based on polymers, Matrigel, a cell secreted protein mixture, has been considered the standard material in terms of cell migration. However, since further development of chip‐based 3D cell culture in cancer research will be largely dependent on the improvement of biomaterials that emulate the ECM and the capacity to scale‐up these complex technologies, Matrigel may not represent the best choice, for several reasons: Its composition is undefined and cannot be fully controlled, changing from batch to batch, with a complex mixture of components (unknown amounts of growth factors and proteins) that may play a role in cell behavior. In terms of practical use, it is not as user friendly as silk, due to its gelling temperature and stiffness/consistency to perform further tests on the hydrogels.^35^, ^36^ Moreover, it will not allow long term experiments, very much needed to understand the real process of tumor and metastasis formation. Development of integrating TE approaches and microfluidics into easy‐to‐use, scalable, reproducible, and cost‐effective systems will be the key to their success and future translation to the market.^37^

The Boyden chamber assay, exploiting a chemokine gradient between upper and lower chambers to drive cell invasion and migration, can be readily adapted to model specific ECM chemistries by simple coating procedures and is undoubtedly a precious tool. However, a comprehensive assessment of 3D tumor cell invasion or migratory mechanisms using histological techniques is difficult to achieve in these systems, with metrics restricted to an end‐point summation of cell numbers in the lower chamber. Controlling the spatial distribution of cells on extracellular proteins at the 2D interface is challenging and we anticipate that with scientific research development, biomaterial platforms with controlled *x, y*, and *z* internal dimensions will prove a valuable improvement. Additionally, the developed 3D dynamic model presents several advantages such as the microchannels in contact with the 3D microenvironment, flow, the real‐time tracking of cells, a fine balance between complexity and experimental control and the ability to isolate the effect of variables. Horseradish crosslinked hydrogels represent a suitable ECM like material, with the advantage of being easily tunable in order to best match the desired application. However, this model still lacks some aspects in order to represent the real complexity of tumor microenvironment. For instance, the use of primary tumor cells (making it a patient specific assay when testing drugs) and a confluent monolayer of endothelial cells along the microchannel will add the very much needed complexity of the cell–cell junctions between endothelial cells and the ECM that they produce, being this our next goal.

## 4 Conclusions

3D in vitro dynamic models allow scientists to study aspects of the tumor microenvironment using specific extracellular matrices and cell types. Controlling and understanding the effect of the various components of these models pushes the field forward and enables investigation of interactions within the tumor microenvironment, as well as the response to stimuli such as chemoattractants and chemotherapeutics. However, microfluidic models need standardization and reproducible formats suitable for high‐throughput applications. If cell migration studies are to be used to test drug efficacy in this context, 3D models have to be reliable with regard to fabrication and incorporation of the most adequate ECM like material to reduce arbitrary cell migration patterns.

We explored for the first time silk hydrogels as ECM like materials to mimic tumor microenvironment and to study colorectal cancer migration, compared to a control group: Matrigel. Moreover, after comparing the proposed 3D dynamic microfluidic model to the traditional Boyden chamber assay, fundamental differences in terms of colorectal cancer cell migration were shown. Ultimately, we developed a tunable Silk hydrogel for the development of a CRC Extravasation 3D Model on a Chip. This model represents a valuable tool to better understand cell migration and tumorigenesis processes, consisting in a proof of concept, where different materials can be assessed as ECM like materials, as well as the influence of chemoattractants.

## 5 Experimental Section

*Materials* *and Reagents*: Cocoons of *Bombyx mori* were provided by the Portuguese Association of Parents and Friends of Mentally Disabled Citizens (Portugal).

*Preparation of Silk Fibroin Hydrogels—Preparation of Silk Fibroin Solution*: The purified silk fibroin (SF) was prepared as described previously by Yan et al.^14^ Briefly, SF was dissolved in lithium bromide (9.3 m), followed by dialysis against distilled water for 48 h before concentrating it using a 20 wt% poly(ethylene glycol) solution. SF solution of 16 wt% concentration was used for further hydrogel preparation.

*Preparation of Silk Fibroin Hydrogels—Composition and In Situ SF Hydrogel Formation*: Hydrogels were prepared by mixing the concentrated SF solution with PBS at appropriate volumes to make final 1%, 2%, and 3% silk solutions. To promote the eSF hydrogel formation, 1 mL of SF solution was mixed with Horseradish Peroxidase (HRP) (100 µL) and hydrogen peroxide (H_2_O_2_) (65 µL) solutions in an eppendorf in a water bath at 37 °C, as optimized previously by the group.^14^ HRP solution (0.84 mg mL^−1^) and H_2_O_2_ (0.36 wt%) were both prepared in PBS. For the outward cell migration (modified Boyden chamber assay), 2% and 3% eSF hydrogels (10 µL) were placed on top of the fluoroblock membrane (VWR, Portugal) and allowed to crosslink for another 20 min. For the 3D dynamic migration assays, 2% and 3% eSF hydrogel discs were prepared by adding 30 µL of the mixture solutions in a 6 mm diameter silicon mold and placed at 37 °C for crosslinking during 20 min. This mold size was used for the crosslinked hydrogel to fit in the Vena4 microwells. For the eSF hydrogels supplemented with hVCAM‐1 (10%) (Prepotech, Portugal), the solution was mixed with hVCAM‐1 (20 µg mL^−1^) and then placed in silicon molds for crosslinking for 20 min. Afterward, hydrogels were taken out of the molds, placed in Vena4 microwell and sealed.

*Preparation of Matrigel Coating and Hydrogel Matrices—Composition and In Situ Hydrogel Formation*: Matrigel coating in outward cell migration (modified Boyden chamber) were reconstituted from Matrigel (BD Biosciences) and diluted (250 µg mL^−1^ in 0.01 m Tris) (pH 8.0), NaCl (0.7%) (Laborspirit, Portugal), according to manufacturer's instructions. Diluted Matrigel was carefully added to the top of the membrane and incubated at 37 °C for 2 h. Then, the remaining liquid (coating buffer) was carefully removed from the permeable support membrane without disturbing the layer of Matrigel. For the 3D hydrogels, Matrigel were added to the 6 mm diameter silicon molds (30 µL) and placed at 37 °C for 30 min. For the hydrogels supplemented with hVCAM‐1 (10%), Matrigel was mixed with hVCAM‐1 and then placed in silicon molds for crosslinking for 30 min.

*Characterization of Hydrogels—Mechanical Properties Determination*: The 1%, 2%, and 3% eSF and Matrigel hydrogels' storage and loss moduli were evaluated by using an oscillatory model in a rheometer (Kinexus Prot, Malvern). For each measurement, SF solution (1 mL) was mixed with HRP (100 µL) and H_2_O_2_ (65 µL), and then the mixture (100 µL) were transferred into 8 mm silicon molds at 37 °C. After crosslinking, hydrogels were placed into the rheometer for evaluation. All samples were assayed using a plate–plate geometry. The measurements were conducted at 37 °C (*n*  =  3). Stress sweeps (0.1 Hz) were first performed to determine LVR for all the tested conditions. Frequency sweeps (0.01–1 Hz) were then performed within the LVR. The values of the shear storage modulus (*G*′) presented in Table 1 were obtained at a frequency of 0.1 Hz.

*Cell Culture*: HCT‐116 cells (human colon cancer cell line) originally obtained from the American Collection of Cell Cultures (ATCC, USA) were used. Cells were continuously grown in DMEM, (Sigma, Germany) supplemented with 10% fetal bovine serum and 1% penicillin and streptomycin under standard conditions (37 °C in a humidified atmosphere containing 5% CO_2_). Medium was changed twice a week and subcultures of cells were performed when confluence reached values of ≈90%.

*Outward Cell Migration (Modified Boyden Chamber Assay)*: Cell migration was analyzed using HTS Corning FluoroBlok Cell Culture Inserts (24 well), with an 8 µm pore size (Becton Dickenson, EUA).

First, the eSF hydrogels and Matrigel coating were performed as described before. Then, HCT‐116 cells were prelabeled with Cell Tracker Red (Invitrogen, Portugal) for 20 min (5 × 10^−3^ m), and then seeded on top of the coating/hydrogels (30 000 cells per well). In the upper chamber, either DMEM medium with 10% v/v FBS or Serum‐free medium were added (300 µL). In the lower chamber, either DMEM medium with 10% v/v FBS, DMEM supplemented with 10% v/v hVCAM or with 30% v/v FBS were added (500 µL). At different time points (0, 2, 5, 24, and 48 h), fluorescence intensity from the bottom was measured using a microplate spectrofluorimeter (BioTek, Portugal) in area‐scan bottom‐reading mode, at excitation/emission wavelengths of 553/570 nm. Results are presented as the increase in fluorescence in relation to the time point 0 h. Fluorescence images of migrating cells were collected using an inverted fluorescence microscope (Leica, Germany). In the last time point (48 h), cells were fixed with formalin (10%) (Sigma, Germany) and stained for F‐actin filaments of the cytoskeleton and nuclei with Texas Red‐X phalloidin (Molecular Probes, Invitrogen, USA) and with 4,6‐diamidino‐2‐phenylindole, dilactate (DAPI blue, Molecular Probes, USA), respectively, following supplier's protocol.

*In Vitro Studies under a 3D Microfluidic Platform—Vena4 Microfluidic Chip*: The Vena4 biochip from Cellix (Ireland) is constituted by 4 channels with 100 µm of height, 800 µm of width, and 2.8 cm of length. Each channel is connected to two microwells (one in each end) where a microfluidic recirculating pump controlled by an iPod Touch (Kima pump) is connected, and a microwell separated by a 8 µm pore size membrane. The sample volume of each channel was 10 µL. The chip was fabricated in optically clear acrylic with a substrate thickness of 500 µm, enabling the observation of cells under a brightfield, fluorescence (Leica, Germany) and confocal microscope (Zeiss, Germany).

*In Vitro Studies under a 3D Microfluidic Platform—Cell Migration Assay in 3D VenaT4 Biochips*: Cell migration under a dynamic 3D microfluidic platform was conducted using VenaT4 biochips (Cellix, Ireland). Each biochip channel was coated with fibronectin (Sigma, Germany) (100 µg mL^−1^) and placed in a humidified sterile Petri dish and incubated at 37 °C for 1.5 h. After the incubation period, HCT‐116 cells were prelabeled with red cell tracker CM‐DiI Dye (1 × 10^−6^ m) (Invitrogen, Portugal) and incubated at 37 °C for 30 min. After, labeled cells were seeded on Vena4 microchannels using a standard yellow pipette (30 000 cells/channel). In order to prevent drying, after the initial 30 min. of incubation, complete medium DMEM was added to the channels. The hydrogels (3% and 2% eSF and Matrigel), previously crosslinked in the silicon molds, were placed in the microwell and sealed. At determined time points (0 and 48 h), the chip was observed under confocal laser scanning microscopy (Leica, Germany) to monitor prelabeled cells' migration toward the hydrogel with and without hVCAM‐1.

*In Vitro Studies under a 3D Microfluidic Platform—ATP Quantification*: To quantify the viable cells that migrated toward the hydrogel, ATP was measured using CellTiter‐Glo Luminescent Cell Viability Assay (Promega, Portugal). For that, hydrogels were removed from Vena4 Biochips after 48 h of culture and placed in 96 well plates. Then, the analysis was performed following the manufacturer's instruction. eSF and Matrigel hydrogels were used as controls. Briefly, CellTiter‐Glo Reagent agent was added (150 µL) to each well of the 96‐well plate and incubated for 10 min at room temperature to lysate cells. Then, the cell lysate (100 µL) was transfer to wells of a 96‐well white opaque microtiter plate (in triplicate). This plate was loaded into the luminometer (Perkin‐Elmer, EUA). The signal intensity of the samples was measured. Light output was given as the integral relative light units (RLUs). ATP measurements were carried out at room temperature. An ATP standard curve was generated using ATP solutions ranging from 5 to 1000 cells and luminescence readings from experimental samples were fit into this curve to generate moles of ATP.

*In Vitro Studies under a 3D Microfluidic Platform—Live/Dead Assay*: The viability of cells migrated into the hydrogels was assessed using the Live/Dead assay. Cell‐laden matrices were washed three times with PBS (Sigma, Germany), then incubated (10 min, 37 °C in the dark) with calcein AM (1 × 10^−6^ m, live cells) and propidium iodide (PI, 1.5 × 10^−6^ m, dead cells) and washed again. Samples were imaged by confocal laser scanning microscopy. Two filters were used: for green excitation/emission (495/515 nm) and for red excitation/ emission (510/595 nm).

*Statistical Analysis*: Statistical analyses were performed using GraphPad Prism 5.0 software version 5.0a. The nonparametric Mann–Whitney test was used to compare two groups, whereas comparison between more than two groups was performed using the Kruskal–Wallis test followed by Dunn's comparison test. A value of *p* < 0.05 was considered statistically significant.

## Conflict of Interest

The authors declare no conflict of interest.

## Supporting information

Supplementary
