# Supplementary material for: Tuning Enzymatically Crosslinked Silk Fibroin Hydrogel Properties for the Development of a Colorectal Cancer Extravasation 3D Model on a Chip
Source: Glob Chall. 2018 May 24;2(5-6):1700100. doi: 10.1002/gch2.201700100 (PMC6607308; doi:10.1002/gch2.201700100)
Supplement: Supplementary file 1 — Supplementary [file GCH2-2-1700100-s001.pdf]

Copyright WILEY-VCH Verlag GmbH & Co. KGaA, 69469 Weinheim, Germany, 2018.

Global Challenges
Open Access

Supporting Information

for *Global Challenges,* DOI: 10.1002/gch2.201700100

Tuning Enzymatically Crosslinked Silk Fibroin Hydrogel Properties for the Development of a Colorectal Cancer Extravasation 3D Model on a Chip

*Mariana R. Carvalho, Fátima Raquel Maia, Sílvia Vieira, Rui L. Reis, and Joaquim M. Oliveira**

7. Supplementary Material

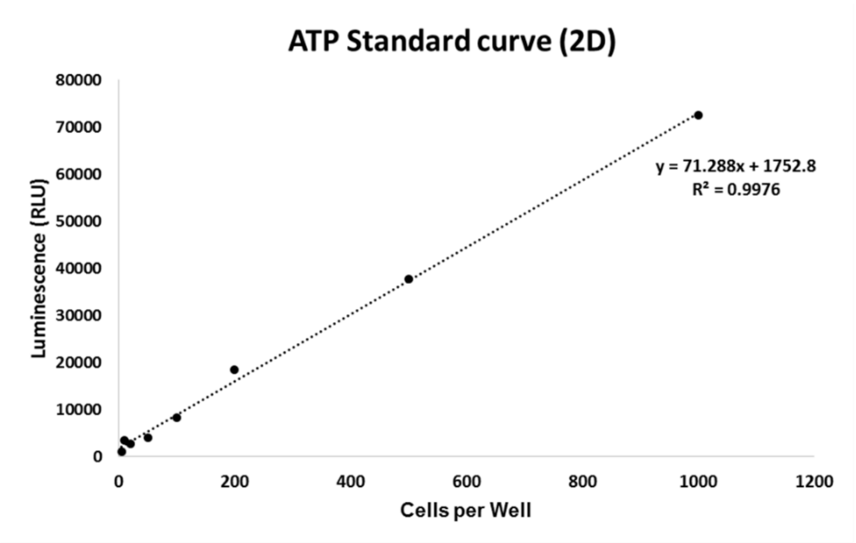

Cell viability of HCT-116 by quantification of ATP. Standard curve generated using ATP solutions ranging from 5 to 1000 cells in 2D culture plates.
